# Integration of deep intronic and RNA sequencing enhances molecular diagnosis in genetically unsolved Pompe cases

**DOI:** 10.1016/j.ymgmr.2026.101307

**Published:** 2026-03-17

**Authors:** Huseyin Onay, Deniz Kor, Fatma Derya Bulut, Ezgi Irmak Burgac, Bahadir Onay, Irem Kaplan, Halise Neslihan Onenli Mungan

**Affiliations:** a Multigen Genetic Diseases Diagnosis Center, Izmir, Turkey; b Department of Pediatrics, Pediatric Metabolism and Nutrition Department Cukurova University, Faculty of Medicine, Adana, Turkey; c Gene2Info Sag.Hiz., Istanbul, Turkey

**Keywords:** Pompe disease, Whole gene sequencing, Alternative splicing, Diagnostic algorithm, RNA sequence analyses

## Abstract

We describe a diagnostic workflow integrating deep intronic and RNA sequencing to resolve genetically unsolved Pompe cases. A five-year-old girl with hypertrophic cardiomyopathy, muscle weakness, recurrent respiratory tract infections, elevated CK levels, and low alpha-glucosidase enzyme activity was referred to the metabolism department. Genetic tests for Pompe disease, conducted by two different genetic laboratories, failed to detect any mutation. Two years later, the patient was referred for further genetic testing. As the third-step genetic test in our diagnostic algorithm, following exonic sequencing and MLPA, the *GAA* whole gene sequencing test was performed and revealed the homozygous NM_000152.5: c.1327–419 A > G variant. RNA sequencing confirmed exon extension, showing that this novel variant created a new donor splice site. Ultimately, the patient was genetically diagnosed and received appropriate treatment. This study highlights the importance of incorporating deep intronic and RNA sequencing is an essential subsequent step in the molecular diagnosis of unsolved Pompe cases, and it further reveals a novel pathogenic variant in the *GAA* gene.

## 1 Introduction

Pompe disease (Glycogen storage disease type II, OMIM: 232300) is an autosomal recessive disorder caused by pathogenic variants in the *GAA* gene, leading to partial or complete deficiency of the lysosomal enzyme acid α-glucosidase (GAA). Skeletal and cardiac muscles are the primary sites of involvement, often leading to hypotonia and muscle weakness. Classic infantile patients, who completely lack *GAA* activity, typically present with hypertrophic cardiomyopathy and severe muscle weakness shortly after birth. Patients with residual enzymatic activity develop later-onset forms, usually classified as childhood or adult onset, which generally lack cardiac involvement [1].

Identification of pathogenic variants in monogenic diseases is crucial for establishing a diagnosis, enabling genetic counselling, and predicting disease severity. Routine diagnostic DNA analysis typically focuses on the coding regions and exon–intron boundaries of *GAA* [1]. This method detects variants in the coding regions and near splice sites but not in the promoter, UTRs, and most of the intronic regions [1].

The prevalence of deep intronic mutations in the *GAA* gene has not been well characterized, mainly because most diagnostic assays are designed to target exonic regions. Some reports suggest that the inclusion of intronic regions in testing can increase the diagnostic yield by approximately 3% through the identification of pathogenic variants [2].

In many Mendelian disorders some patients remain genetically unsolved despite the clear clinical signs and symptoms of the disease. There are several reasons for missing mutations which cannot be detected in routine DNA sequencing; variants located in deep intronic or regulatory regions, or mutations present in a mosaic state. Such unsolved cases require attention, and additional genetic diagnostic techniques must be used to solve. Here, we present a diagnostic strategy for Pompe disease integrating deep intronic sequencing as a third-tier test following exonic sequencing and MLPA.

## 2 Materials and methods

### 2.1 Clinical assessment and sample collection

The patient, a 5-year-old girl, was born to consanguineous parents. At 22 months of age, she was diagnosed with hypertrophic cardiomyopathy during hospitalization for malnutrition, mild muscle weakness, and a lower respiratory tract infection. Notably, there was no reported family history of similar conditions. The patient was delivered via cesarean section at term, with a birth weight of 3000 g. The perinatal history was unremarkable. Neuromotor developmental milestones were appropriate for her age, including head control at 2 months, crawling at 7 months, first words at 9 months, independent walking at 14 months, and toilet training at 30 months. On initial physical examination, the patient's weight was 8 kg (−2.83 standard deviation score [SDS]), height was 78 cm (−2.07 SDS), and head circumference was 45 cm (−1.95 SDS). She exhibited a myopathic facial appearance and mild hepatomegaly, with the liver palpable 3 cm below the costal margin. The patient was able to walk independently but displayed mild hypotonia. Electrocardiography (ECG) revealed a shortened PR interval and increased QRS voltage in the precordial leads. Echocardiography demonstrated hypertrophic cardiomyopathy, with an interventricular septum thickness of 10 mm, and mild (grade I) mitral insufficiency. Creatine kinase (CK) levels were elevated, ranging from 224 to 684 U/L (normal range: 28–204 U/L). Alpha-1,4 glucosidase activity measured via dried blood spot was significantly reduced on two separate tests: 0.7 μmol/l/h (normal >3.3) and 0.3 μmol/l/h (normal >2.0). Initial *GAA* gene sequencing in 2 different genetic laboratories revealed no mutation. These steps also repeated in our lab (*GAA* exonic sequencing as the first step and MLPA analysis as the second step of the algorithm) did not reveal any pathogenic variants. For the further analysis, peripheral blood samples were collected from the patient, her parents, and her four siblings. Informed consent was obtained from the family for all genetic testing procedures and publication purposes.

### 2.2 Genetic studies

Genomic DNA has been obtained from the blood lymphocytes using the QIAamp DNA Blood Mini QIAcube Kit (Qiagen, Hilden, Germany) with the manufacturer's protocols. *GAA* whole gene was amplified using the *GAA* Whole Gene Sequencing Kit (Multigen, Izmir, Turkey). Sequencing was performed with the MiSeq Reagent Kit V2 on the MiSeq desktop sequencer (Illumina, San Diego, CA). Analysis was performed with Integrative Genomics Viewer (IGV) software (Broad Institute, Cambridge, MA). The variants visualized with IGV were controlled in databases of HGMD® and ClinVar. American College of Medical Genetics (ACMG) guidelines were followed for variant pathogenicity classification [3]. Familial segregation was performed for the mother, father, and four other healthy siblings in the same manner as for the index case following the detection of the suspected variant (Fig. 2b). RNA isolation was performed using the Monarch Total RNA Miniprep Kit from the patient's peripheral blood sample. cDNA was synthesized from the isolated RNA using the Qiagen QuantiTect Reverse Transcription Kit. cDNA was amplified by polymerase chain reaction using primers (Table 1) designed for exons 7, 8, 9 and 7–8. All amplicons were analyzed via agarose gel electrophoresis (Fig. 4). Subsequently, amplicons were sequenced using the MiSeq Reagent Kit V2 on the MiSeq desktop sequencer (Illumina, San Diego, CA). The analysis was performed with the Integrative Genomics Viewer (IGV) software (Broad Institute, Cambridge, MA) (Fig. 3), STAR was utilized as alignment tool and output BAM files indexed using Samtools.

**Table 1:** Primers for cDNA exon7,8 and 9.

Forward	CGTTCATGCCGCCATACT
Reverse	GGTCTCGTTGGTGATGAAAAC

## 3 Results

### 3.1 Genetic findings

The *GAA* whole gene sequencing test identified a deep intronic variant, c.1327–419 A > G, in a homozygous state within intron 8 of the *GAA* gene (NM_000152.5). This variant was not detected in prior exonic analyses due to its deep intronic location. It was absent in population databases such as gnomAD, meeting the PM2 criterion for pathogenicity. SpliceAI predicted the variant to be deleterious, fulfilling the PP3 criterion. Based on ACMG guidelines, the variant was classified as a VUS.

### 3.2 Family segregation analysis

Segregation analysis revealed that the mother, father, and three siblings were heterozygous carriers of the variant, while one sibling was unaffected (Fig. 2b).

### 3.3 RNA analysis

Agarose gel electrophoresis showed elongation of exon 8 in the patient. The father, identified as a heterozygous carrier, displayed two PCR product bands: one corresponding to the normal transcript and the other to the variant allele. IGV analysis of RNA sequencing data revealed that exon 8 was abnormally fused with the adjacent intron, confirming abnormal splicing in the patient.

In silico translation of the wild-type and variant cDNA transcripts using the *Expasy Translate* tool demonstrated that inclusion of the 697 bp intronic sequence causes a frameshift, introducing a premature termination codon (TAA) and resulting in early termination of translation (Table 2, Table 3).

**Table 2:** Expected (WT) amino acid sequence:

M M I V D P A I S S S G P A G S Y R P Y D E G L R R G V F I T N E T G

**Table 3:** Variant amino acid sequence:

M M I V V C A P T L W V F G K G A A R C P V A P S L C S V I L V P V W S P R M F S E G L C D I E G I S R S L Q A W P Q L S R E V G F E G P Q K W P G A T Q G S V R C R L L E L P – S K R P G A - - T D V

## 4 Discussion

Pompe disease, caused by mutations in the *GAA* gene, is a lysosomal storage disorder that often presents molecular diagnostic challenges, particularly in cases with strong clinical and biochemical evidence but unremarkable findings in standard genetic testing. The limitations of conventional diagnostic techniques, such as exonic sequencing analysis and MLPA, which primarily target coding regions and proximal splice sites, are well-documented [1], [4]. These approaches often fail to detect variants in deep intronic regions or regulatory sequences, as demonstrated in this case. Yet, intronic mutations may alter canonical splice sites, resulting in exon skipping, intron retention, or pseudo-exon activation, all of which can lead to aberrant mRNA transcripts [5].

In clinical practice, molecular confirmation is often required not only for diagnostic clarification but also for treatment eligibility. In this case, although the patient fulfilled the clinical and biochemical criteria for Pompe disease, initiation of enzyme replacement therapy was delayed for nearly two years due to healthcare reimbursement policies in Türkiye that require molecular genetic confirmation for treatment approval. For this reason, further advancement of the analytical workflow to establish a molecular diagnosis becomes increasingly important.

Intronic sequences proximal to exon-intron boundaries represent the most extensively investigated category of intronic mutations due to their significant impact on splicing and gene regulation [6]. The HGMD database currently lists 805 coding and 121 non-coding variants, with only a few located beyond 20 nucleotides from exon intron boundaries. Recent advances in whole-genome sequencing have revealed an increasing number of pathogenic variants residing deep within intronic regions, far beyond (more than 100 base pairs away) the typical exon-intron boundaries [7]. These discoveries highlight the critical need to understand how deep intronic variants influence pre-mRNA splicing and their potential role in driving disease mechanisms. As a result, genetic research is entering a new era, focusing on the functional consequences of non-coding variants and their contributions to complex phenotypes [7].

**Fig. 1:**
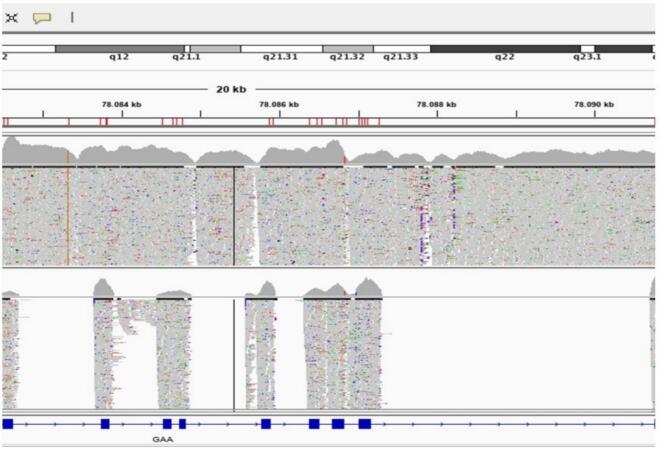
Upper row represents *GAA* Whole Gene Sequencing Test, while bottom row shows the standard exonic *GAA* analysis.

**Fig. 2:**
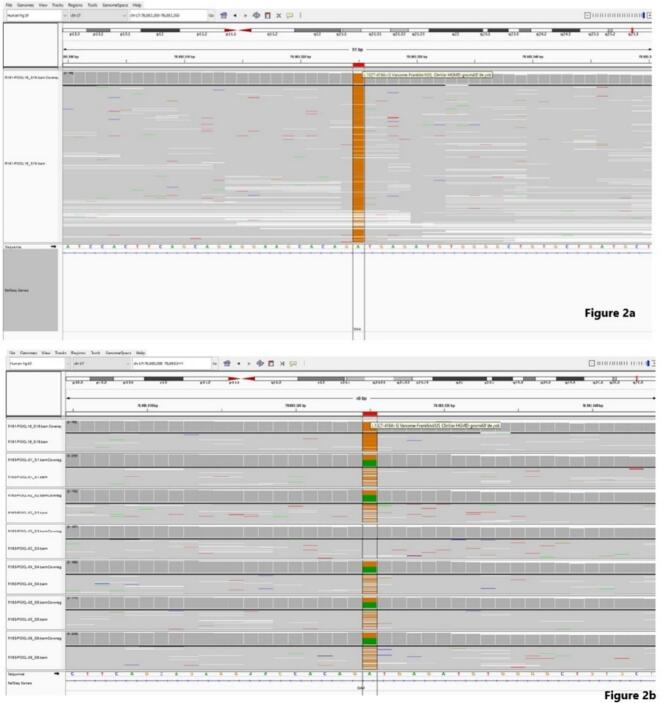
a: In patient, homozygous c.1327–419 A > G variant was detected in *GAA* gene. b: Segregation Analysis: From top to bottom, in order; index case, mother, father, sibling 1, sibling 2, sibling 3, and sibling 4.

In this report, whole *GAA* gene sequencing was implemented as a third-tier test, enabling analysis of exonic, intronic, untranslated, and promoter regions. Following it, RNA sequencing was applied as a fourth-tier step. As illustrated in this case, the c.1327–419 A > G variant was identified deep within intron 8 of the *GAA* gene and therefore could not be detected by standard exonic sequencing (Fig. 1). This variant is not present in gnomAD or other population databases, thus meeting the PM2 criterion [8]. SpliceAI predicts this variant to be deleterious, thus meeting the PP3 criterion [8]. SpliceAI analysis predicted a high donor gain score (0.88) indicating activation of an alternative 5′ splice site, whereas the acceptor gain score was moderate (0.30). The variant was classified as a Variant of Uncertain Significance (VUS) according to ACMG criteria. The segregation study demonstrated that the variant was present in a homozygous state only in the affected proband. (See Fig. 2.)

**Fig. 3:**
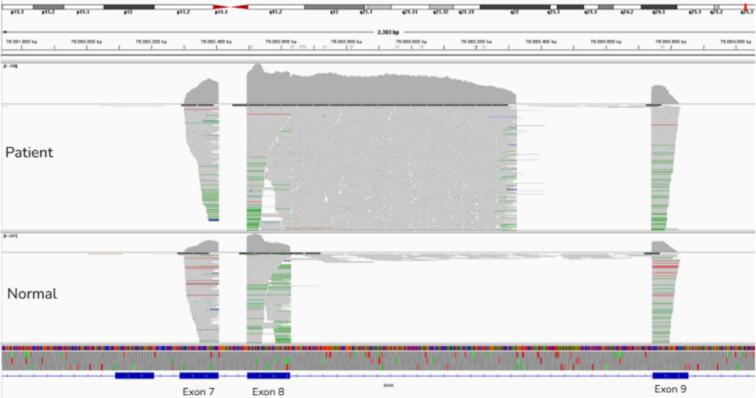
This figure compares the RNA sequencing of the *GAA* gene results of a normal control and a patient sample.In the normal RNA sequencing data, the alignment tracks show expected splicing patterns. In the patient RNA sequencing data, exon 8 is abnormally contiguous with the adjacent intron, indicating *alternative 5′ splice site*. This defect suggests a failure in the splicing machinery to remove the intron between Exon 8 and Exon 9.

**Fig. 4:**
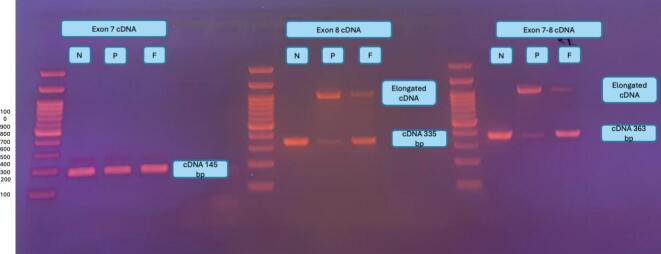
This figure shows the results of gel electrophoresis of cDNA amplicons corresponding to specific *GAA* exons (Exon 7, Exon 8, and Exon 7–8) in samples from a control (N), patient (P), and father (F). The cDNA fragment corresponding to Exon 7 is identical across all samples (control, patient, and father), with a product size of 145 bp. The Exon 8 cDNA is abnormally elongated in patient, as evidenced by a larger fragment size. This confirms the presence of *alternative 5′ splice site*, resulting in an elongated product. In the father (F), who is identified as a carrier, two bands are observed. The presence of both bands (normal and elongated) indicates heterozygosity in the father, with the normal allele being more abundantly expressed.

To investigate the functional impact of the variant, an RNA study was planned as detailed in the method section. RNA was isolated from the blood samples of the control (N), patient (P), and father (F). The *GAA* exons were amplified using appropriate primers. The amplified products were analyzed by gel electrophoresis. The amplicons corresponding to exon 7 were identical in the control, patient, and father. However, exon 8 was found to be abnormally elongated in the patient. In the father, identified as a carrier, the normal amplicon was more abundant, but the amplicon synthesized from the variant allele was also present (Fig. 4). Moreover, a faint band corresponding to cDNA amplicons of exon 7 and exons 7–8 was observed in the patient sample (Fig. 4). The presence of a faint band corresponding to a normal or near-normal transcript in the patient sample suggests that the constitutive 5′ splice site may still be partially utilized, at least in peripheral blood cells. However, this observation should be interpreted with caution, as splicing patterns in blood may not fully reflect those in affected tissues such as muscle. Nevertheless, even low levels of residual normal transcript expression could potentially contribute to a less severe clinical presentation. The extent to which this residual splicing activity influences disease severity remains uncertain and warrants further investigation in disease relevant tissues. The RNA products were sequenced, revealing that exon 8 was contiguous with the adjacent intronic sequence (Fig. 3), with no evidence of pseudoexon inclusion on RNA sequencing. Although sequencing of the normal-sized transcript band could not be performed, the patient's juvenile-onset phenotype together with detectable residual GAA enzyme activity is consistent with the presence of residual normal transcript expression.

Intron retention occurs when an intron, which is typically removed during mRNA splicing, remains included in the mature mRNA transcript [9]. Partial intron retention may result from the activation of alternative 5′ or 3′ splice sites. This can result from splice site mutations, changes in regulatory regions, or disruptions in trans-acting splicing factors. In this case, the c.1327–419 A > G variant likely causes abnormal splicing by introducing an alternative 5′ splice site, leading to incomplete intron removal and its partial retention in the mRNA.

The consequences of partial intron retention can be significant. It may lead to the inclusion of a premature stop codon within the retained intron, resulting in truncated protein products. Alternatively, the abnormal transcript might be targeted for degradation through mechanisms like nonsense-mediated decay (NMD) [7]. In some cases, abnormal splicing can produce alternative protein isoforms or render the protein nonfunctional. However, it has also been identified as a physiological mechanism of downregulation, and recent studies have shown that intron retention may not be solely related to short product lifespan [7], [9].

This case illustrates the diagnostic complexity of Pompe disease, particularly in patients with deep intronic variants. These analyses revealed the impact of the c.1327–419 A > G variant on splicing, demonstrating the creation of an alternative 5′ splice site and its potential to disrupt normal GAA enzyme function. This case contributes to the literature by highlighting the role of deep intronic mutations in disease mechanisms, emphasizing the importance of advanced genomic techniques and RNA analysis in diagnosing and understanding Pompe disease, and raising awareness about these complex genetic factors.

## CRediT authorship contribution statement

**Huseyin Onay:** Writing – review & editing, Writing – original draft, Supervision, Software, Resources, Methodology, Investigation, Formal analysis, Data curation, Conceptualization. **Deniz Kor:** Writing – review & editing, Writing – original draft, Data curation. **Fatma Derya Bulut:** Writing – review & editing, Writing – original draft, Investigation, Conceptualization. **Ezgi Irmak Burgac:** Writing – original draft, Supervision, Methodology, Formal analysis. **Bahadir Onay:** Writing – review & editing, Writing – original draft, Methodology, Investigation. **Irem Kaplan:** Writing – original draft, Supervision, Methodology, Formal analysis. **Halise Neslihan Onenli Mungan:** Writing – review & editing, Supervision.

## Declaration of competing interest

The authors have declared no competing interests.

## Data Availability

Data will be made available on request.
